# Time-Elastic Generative Model for Acceleration Time Series in Human Activity Recognition

**DOI:** 10.3390/s17020319

**Published:** 2017-02-08

**Authors:** Mario Munoz-Organero, Ramona Ruiz-Blazquez

**Affiliations:** Telematics Engineering Department, Universidad Carlos III de Madrid; Av. Universidad, 30, 28911 Leganes, Spain; munozm@it.uc3m.es

**Keywords:** human activity recognition, accelerometer sensors, auto-encoders, generative models for training deep learning algorithms

## Abstract

Body-worn sensors in general and accelerometers in particular have been widely used in order to detect human movements and activities. The execution of each type of movement by each particular individual generates sequences of time series of sensed data from which specific movement related patterns can be assessed. Several machine learning algorithms have been used over windowed segments of sensed data in order to detect such patterns in activity recognition based on intermediate features (either hand-crafted or automatically learned from data). The underlying assumption is that the computed features will capture statistical differences that can properly classify different movements and activities after a training phase based on sensed data. In order to achieve high accuracy and recall rates (and guarantee the generalization of the system to new users), the training data have to contain enough information to characterize all possible ways of executing the activity or movement to be detected. This could imply large amounts of data and a complex and time-consuming training phase, which has been shown to be even more relevant when automatically learning the optimal features to be used. In this paper, we present a novel generative model that is able to generate sequences of time series for characterizing a particular movement based on the time elasticity properties of the sensed data. The model is used to train a stack of auto-encoders in order to learn the particular features able to detect human movements. The results of movement detection using a newly generated database with information on five users performing six different movements are presented. The generalization of results using an existing database is also presented in the paper. The results show that the proposed mechanism is able to obtain acceptable recognition rates (*F* = 0.77) even in the case of using different people executing a different sequence of movements and using different hardware.

## 1. Introduction

Human Activity Recognition (HAR) based on low-level sensor data is widely used in order to provide contextual information to applications in areas such as ambient-assisted living [1], health monitoring and management [2], sports training [3], security, and entertainment [4]. Raw data from sensors such as accelerometers, Global Position System (GPS), physiological sensors, or environmental sensors are processed into movement-related features that are used to train machine learning algorithms that are able to classify different activities and movements [4]. By detecting the particular activity that a user is performing at each particular moment, personal recommender systems could be used in order to provide feedback and advice to increase the user’s wellbeing, optimize the user tasks, or adapt the user interface in order to optimally convey information to the user. 

Different variants exist in previous research studies in order to recognize human activities from sensor data. When focusing on the type of sensor used, the recognition of human activities has been approached in two major different ways, namely using external and wearable sensors [4]. When approaching the subject based on the way that is used in order to segment the sensed data, two major alternatives have been previously used to recognize human activities, namely temporal windows of sensed data and activity composition by combining the detection of sporadic atomic movements [5]. Depending on the way in which the computed features are defined, two major approaches exist, either using hand-crafted features or automatically learning the optimal features from data [6]. Each approach has desirable and undesirable aspects. Environmental sensors such as video cameras do not require the user to wear the sensors and therefore are less intrusive but are restricted to particular areas and raise privacy issues. Windowed approaches are simple to implement but only adapt to activities that can be fully statistically described in the duration of the time window and do not overlap or are concatenated with other activities during that temporal window. Hand-crafted features can introduce a priori models for the sensed data for the execution of particular activities but require expert knowledge and tend not to generalize among different applications. Deep learning approaches are able to automatically detect the optimal features but require bigger training datasets in order to avoid overfitting problems [7]. 

In this paper, we propose a novel mechanism that focuses on the desirable aspects of previous proposals in order to recognize human activities while trying to minimize the undesirable aspects. In order to detect each particular activity, candidate temporal windows of sensed data are pre-selected based on basic statistical properties of adjacent points of interest (such as consecutive local maximum acceleration points or consecutive local maxima in the standard deviation of the acceleration time series). Time windows are selected and aligned according to the pre-selected points of interest. A stack of auto-encoders is then used in order to assess if a candidate window of sensed data corresponds to a particular human activity or movement. The stack of auto-encoders is trained with similar temporal windows obtained from performing the activity at different speeds by different people. In order not to require an extensive training phase and data gathering process, a generative model has been defined that takes into account the elastic temporal variation of the sensed data when performing the same activity at different speeds. The model is able to generate data for intermediate speeds based on a limited number of training samples. Each human activity or movement to be detected is assessed by a separately trained stack of auto-encoders. The Pearson correlation coefficient is used in order to compute the similarity of each pre-selected data segment with each activity to be recognized. 

The rest of the paper is organized as follows. Section 2 captures the previous related studies and motivates the research in this paper. Section 3 describes the proposed generative model in order to estimate missing data samples in the data gathering process so as to provide enough statistical information to the detection algorithm to characterize a particular movement. The model uses the elastic deformation of the time series when performing the same movement at different speeds. Section 4 details the architecture of the proposed detection algorithm and the way in which it is trained. The detection algorithm is based on a stack of auto-encoders. Section 5 presents the results for detecting two particular movements in a new database that we have created using five people executing six different movements. Section 6 is dedicated to assessing the generalization of results by applying the algorithm trained using our database to the data in a different database in which the participants executed one common movement. Finally, Section 7 captures the major conclusions of the paper. 

## 2. Human Movement Detection from Accelerometer Sensors

Human Activity Recognition has been approached by different research studies in different ways since the first contributions in the late 1990s [8], either using environmental sensors such as cameras [9] and smart environments [10] or wearable sensors such as accelerometers and gyroscopes [11]. Body-worn sensors are preferred in order to preserve privacy and continuously monitor the user independently of his or her location [4].

Body-worn sensors provide one or multiple series of temporal data depending on the activity that the user is performing. In order to extract the particular activity from the sensed data, different machine learning algorithms have been used over some features computed from selected segments of raw data. Different features could be defined in order to better capture the statistical differences among the activities to be detected in either the time or frequency domain [4]. Instead of using hand-crafted features, features could be learned from data, using an optimization process based on some pre-defined features in order to select the most important ones to detect a particular activity [12], combine or project pre-defined features in order to reduce the number of features [13], or use deep learning machine learning techniques to automatically generate the best features for a particular dataset [14].

An example of applying machine learning techniques over hand-crafted features for Human Activity Recognition (HAR) can be found in [15]. The authors used Decision Trees, Random Forest (RF), Naive Bayes (NB), Support Vector Machines (SVM), and K-Nearest Neighbors (KNN) machine learning algorithms applied to acceleration data to compare detection rates when attaching the accelerometer to different parts of the body (wrist, elbow, chest, ankle, knee, and belt). The authors used several hand-crafted features over windowed data and a movement detection algorithm based on an empirical threshold applied to the differences between consecutive values of the lengths of the acceleration vector. 

Hand-crafted features could be applied to windowed segments of sensor data or be based on particular relevant points of the time series of sensed data instead, such as in [5], in which the authors use statistical properties of adjacent maximum and minimum values in acceleration signals in order to recognize different movements, taking into account the energy constrains for in-sensor algorithms.

Deep learning architectures are able to automatically learn the best features to be used for a particular dataset. Plötz et al. [16] present a general approach to feature extraction based on deep learning architectures and investigate the suitability of feature learning for activity recognition tasks. Avoiding hand-crafted features lessens the burden for application-specific expert knowledge. Gjoreski et al. [17] compare hand crafted features based machine learning algorithms such as Random Forests, Naïve Bayes, k-nearest Neighbor, and Support Vector Machines with deep learning architectures based on a convolutional neural network (CNN), showing that the latter was able to outperform the recognition results by at least two percentage points. Yang et al. [6] performed a similar analysis by introducing a convolutional neural network (CNN) for Human Activity Recognition (HAR). The key advantages of using a deep learning architecture are: (i) feature extraction is performed in task-dependent and non-hand-crafted manners; (ii) extracted features have more discriminative power w.r.t. the classes of human activities; (iii) feature extraction and classification are unified in one model so their performances are mutually enhanced. The proposed CNN method outperformed other state-of-the-art methods such as k-nearest Neighbor and Support Vector Machines in classification for the HAR problems.

A review of unsupervised feature learning and deep learning for time-series modeling in general is presented in [14]. Restricted Boltzmann Machines, Auto-encoders, Recurrent Neural Networks, and Hidden Markov Models are described and how these machine learning techniques can be applied to time series obtained from sensors.

The authors in [7] use a deep recurrent neural network (DRNN) architecture in order to recognize six different human activities (“stay”, “walk”, “jog”, “skip”, “stair up”, and “stair down”) using a combination of a tri-axial accelerometer and a gyroscope. If the dataset is divided into segments of raw data containing a single activity, the optimal training parameters are able to detect the correct activity with a 95.42% rate. However, if the algorithm is applied to non-pre-segmented data, the recognition rate decreases to 83.43% for the best parameters and when the training and validation is performed using the same dataset. The optimal number of layers in the DRNN was found to be 3. The more layers we add to a deep learning architecture, the more degrees of freedom we have and therefore the easier it is to achieve higher detection rates for a particular dataset. However, the generalization of results when validating with a different dataset may decrease due to overfitting problems. 

The research study in [18] uses a deep convolutional neural network to perform Human Activity Recognition (HAR) using smartphone sensors by exploiting the inherent characteristics of activities and 1D time series signals, at the same time providing a way to automatically and data-adaptively extract robust features from raw data. The authors recognize six different activities (“walking”, “stair up”, “stair down”, “sitting”, “standing”, and “laying”) using a 2.56-s window (with 50% overlap) for the six-dimensional raw sensed data from a tri-axial accelerometer and a gyroscope. The experimental results show that a three-layer architecture is enough to obtain optimal results. The best results show an overall performance of 94.79% on the test set with raw sensor data that are similar to those obtained using Support Vector Machines (SVM).

Wang et al. [19] make use of auto-encoders for the recognition of Activities of Daily Life (ADL) based on sensed information from environmental sensors. The results outperformed previous machine learning algorithms based on hand-crafted features such as Hidden Markov Models, Naïve Bayes, 1-nearest Neighbor, and Support Vector Machines. 

We will use a similar approach based on stacked auto-encoders applied to one of the axes of a single accelerometer placed on the wrist of the dominant hand. In order to simplify the data gathering for the training phase, a novel generative model based on the time elasticity of the human movements when performed at different speeds is added. A different stack of auto-encoders is trained for each activity to be detected. A simple pre-detection algorithm based on the statistical properties of some points of interest for each activity is added in order to pre-select candidate time windows and a time-offset cancellation mechanism (sequence alignment) is used by centering the selected window based on the detected candidate points of interest.

## 3. Elastic Generative Models for Movement Description

Human activities can be described as a sequence of movements that can be executed at different speeds. In order to characterize each movement, we will use a generative model to parameterize the elasticity of the sensed time series performed at different speeds. 

Let us define $s_{i}(t)$ as the time series capturing the sensed data for a particular execution of a particular movement performed at speed $v_{i}$. The relationship between two different executions of the same movement at different speeds can be modeled as described by Equation (1):

(1) $$s_{i}\left(t-t_{c}\right)=\alpha_{ij}(t-t_{c})\cdot s_{j}\left(\beta_{ij}\left(t-t_{c}\right)\right)$$

(2) $$\beta_{ij}=\frac{v_{i}}{v_{j}},$$

where $t_{c}$ is an alignment factor (we have aligned all the movements to the center of the windowed data), $\beta_{ij}$ is the ratio of execution speeds (as described in Equation (2)), and $\alpha_{ij}(t-t_{c})$ captures the deformation coefficient for a particular value in the time series. If $\alpha_{ij}(t-t_{c})=\alpha_{ij}$ for all values of *t* (does not depend on *t*) the execution of the same movement at different speeds generates scaled versions of the same data. $\alpha_{ij}(t-t_{c})$ is modeled as a function of time (relative to the alignment point). The bigger the value of the variance of $\alpha_{ij}(t-t_{c})$ the larger the deformation of the time series when executing the movement at a different speed. 

A generative model is able to estimate $s_{k}(t)$ when other samples are at different speeds. Let us assume that we have two samples at speeds $v_{i}$ and $v_{j}$ and that $v_{i}<v_{k}<v_{j}$. The time series for $s_{k}(t)$can be written as described by Equations (3) and (4):

(3) $$s_{k}\left(t-t_{c}\right)=\alpha_{ki}(t-t_{c})\cdot s_{i}\left(\beta_{ki}\left(t-t_{c}\right)\right),$$

(4) $$s_{k}\left(t-t_{c}\right)=\alpha_{kj}(t-t_{c})\cdot s_{j}\left(\beta_{kj}\left(t-t_{c}\right)\right),$$

and $\begin{array}{ccc} \alpha_{ki}(t-t_{c})\to 1 & if & v_{k}\to v_{i} \end{array}$ and $\begin{array}{ccc} \alpha_{kj}(t-t_{c})\to 1 & if & v_{k}\to v_{j} \end{array}$. 

We could estimate $s_{k}(t)$ by interpolating from $s_{i}(t)$ and $s_{j}(t)$, as captured in Equation (5), by combining Equations (3) and (4) and approximating $\alpha_{ki}(t-t_{c})$ and $\alpha_{kj}(t-t_{c})$ by $\frac{2(v_{j}-v_{k})}{v_{j}-v_{i}}$ and $\frac{2(v_{k}-v_{i})}{v_{j}-v_{i}}$. These values are chosen so that Equation (5) fulfills the limit cases of $\begin{array}{ccc} \alpha_{ki}(t-t_{c})\to 1 & if & v_{k}\to v_{i} \end{array}$ and $\begin{array}{ccc} \alpha_{kj}(t-t_{c})\to 1 & if & v_{k}\to v_{j} \end{array}$:

(5) $$s_{k}\left(t-t_{c}\right)=\frac{v_{j}-v_{k}}{v_{j}-v_{i}}\cdot s_{i}\left(\beta_{ki}\left(t-t_{c}\right)\right)+\frac{v_{k}-v_{i}}{v_{j}-v_{i}}\cdot s_{j}\left(\beta_{kj}\left(t-t_{c}\right)\right).$$

The model will also need a maximum and minimum value for the speed of execution. These values can be estimated based on the measured samples of sensed data.

## 4. Using a Stack of Auto-Encoders Based on the Generative Model

Auto-encoders use the combination of an encoder and a decoder function in order to try to minimize the reconstruction errors for the samples in the training set. Auto-encoders can be stacked by connecting the reconstructed output of the “layer I” auto-encoder to a “layer I + 1” auto-encoder. Auto-encoders are designed to minimize the error between the input and the reconstructed output according to Equation (6):

(6) $$\varepsilon^{2}\left(x,x^{r}\right)={\left\|x-f_{2}\left(W^{\prime }\left(f_{1}\left(Wx+b\right)\right)+b^{\prime }\right)\right\|}^{2},$$

where $x^{r}$ is the reconstructed signal which is the concatenation of the encoder and decoder functions (*f*_1_ and *f*_2_ are activation functions such as the sigmoid function). A final detection function is required at the end of the stacked auto-encoders in order to assess the similarity of the input and the reconstructed output. We have used the Pearson’s correlation coefficient as a similarity function. 

**Figure 1:**
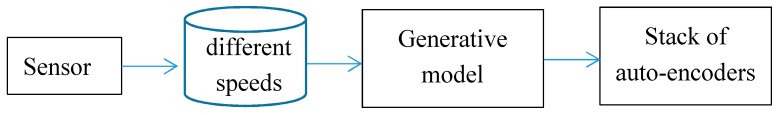
Training phase.

Figure 1 shows the training phase of the system. We select segments of samples from the sensor device containing the particular human movement to be trained. Segments of data are aligned at the center of the selected window (of a particular length in order to contain enough information to describe the movement). The samples are taken executing the movement at different speeds. The generative model described in Section 3 is then used to reconstruct training samples at different speeds in order to properly characterize the human movement when performed at those speeds missing in the captured data. The original data and the newly generated data are then used to train a stack of auto-encoders.

**Figure 2:**
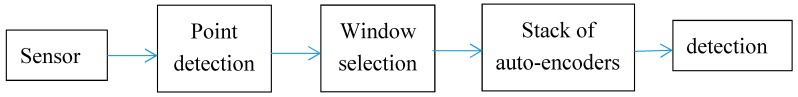
Movement detection.

Figure 2 shows the movement detection process. A new candidate segment of non-labeled data is captured from the sensors. The data are aligned using the same procedure as that used for the training data, using the detection of particular points of interest in the raw signal fulfilling certain properties. The window of input data is then obtained and used as the input for the stack of auto-encoders. The detection phase computes the Pearson’s correlation coefficient and is compared to the values obtained for the same coefficient when using the training data.

The proposed algorithm has some parameters that have to be selected, such as the number of auto-encoders to stack or the number of hidden units at each layer. Section 5 will show the results for the particular cases of detecting the “cutting with a knife” and “eating with a spoon” movements inside acceleration time series containing six different movements (“sitting”, “eating with a spoon”, “cutting with a knife”, “washing the hands”, “walking”, and “free random movements”). A new database for five different people executing the six different movements has been generated. The parameters will be selected based on the ability to detect movement when the movement is performed (recall) and not to detect false positives in segments of data that correspond to other movements (precision). In order to assess the generalization of the algorithm, we will use the trained algorithm with the data in Section 5 in order to detect “cutting” movements in the database in [20] in Section 6 (using different people and different hardware to implement the sensors). 

## 5. Training and Validation Based on a Newly Created Database

A new human activity database combining the repetition of six different movements by five different people has been created in order to train and assess the generative model proposed in the previous section. Ethics approval was obtained through the process defined by the Carlos III of Madrid University Ethics Committee (approved by the agreement of University’s Government Council on 25 September 2014). An individual informed consent to participate in the experiment has been obtained from each participant. The demographics of the five participants are captured in Table 1. 

Each user was wearing a Nexus 6 Android device (which contains a three-axial accelerometer) attached to the wrist of the dominant hand and was asked to repeat the following movements for 1 min each:

1. Idle
2. Eating soup with a spoon
3. Cutting meat
4. Washing hands
5. Walking
6. Free arm movements

**Figure 3:**
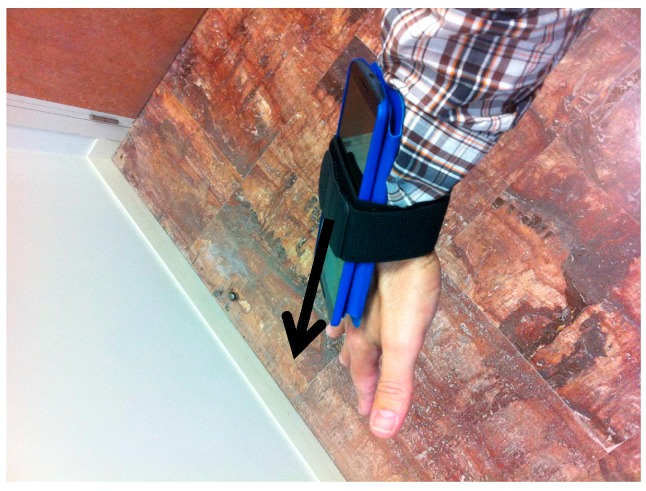
Data gathering system.

The data gathering device used is captured in Figure 3. Although the Nexus 6 Android device is equipped with a three-axial accelerometer, we will use a “worst case” scenario by selecting only one component from the acceleration vector (represented by a black arrow in Figure 3). Using at the same time the data in all the acceleration components is expected to provide even better results that we will study in future work.

The acceleration data were re-sampled at 100 Hz. Each 1-min period contained several repetitions of the same movement. In order to train the stack of auto-encoders, the generative process described in the previous section was used on selected segments of the “cutting with a knife” and the “eating with spoon” movements. Each selected segment contained a window of 2 s of sensed data aligned at the center of the window. The “cutting with a knife” movement constitutes a concatenation of several repetitions (a variable number of times) of a “move forward” and “move backward” movements executed at similar frequencies. Selected time windows of sensor data can be aligned to each central local maximum value in the sequence. In the case of the “cutting with a knife” movement, the speed of execution of each segment $v_{i}$ was estimated as the inverse of the time span between two consecutive maxima in the acceleration data. In the case of the “eating with spoon” movement, each movement of moving the spoon from the plate to the mouth and back to the plate can be described as a concatenation of three gestures (moving up, eating, and moving down). The “up” and “down” gestures can be pre-filtered based on the maximum values for the standard deviation of the acceleration series in a short time window centered at each point. The alignment of each candidate movement will be done to the mean point between the maximum values representing the “up” and “down” movements. In the case of the “eating with spoon” movement, the speed of execution of each movement $v_{i}$ was estimated as the inverse of the time span between the estimated “up” and “down” movements.

The generative model was used on the selected segments of sensed data corresponding to the “cutting” movement in order to generate 51 samples at equally spaced speeds between the minimum and maximum values detected in the sensed samples. In a similar way, 142 samples were generated for the “eating with spoon” movement. The participants were asked to execute the movements at different speeds during the 1-min window. 

The 51 and 142 generated samples were used to train different configurations of parameters for the stack of auto-encoders in order to detect each movement. The trained auto-encoders were then validated using a leave-one-out architecture in which four users were used to train the system and the other one to assess the truly classified samples (the process was repeated leaving out each user and averaging results). In order to compare the classification results provided by each configuration, the obtained precision, recall and *F* score have been used. Defining *tp* as the number of “cutting” or “eating with spoon” movements that are correctly classified, *Tp* as the total number of “cutting” or “eating with spoon” movements present in the validation sequence and *fp* as the total number of “non-cutting” or “non-eating with spoon” movements that are classified as positive samples, the precision, the recall and the *F* score can be defined as:

(7) $$\begin{array}{l} precision=\frac{tp}{\left(tp+fp\right)}\\ recall=\frac{tp}{Tp}\\ F=2\cdot \frac{precision\cdot recall}{precision+recall} \end{array}.$$

The design parameters for the stack of auto-encoders that are going to be assessed are the number of layers (for one, two, and three layers, according to the previous related studies presented in Section 2) and the number of hidden units inside the encode and decode functions of each layer (hidden units). 

The results of previous related studies based on the use of deep learning techniques applied to sensor data in order to detect human movements and activities are captured in Table 2. A final row has been added to capture the results from the analysis of a database that we are going to use to assess the generalization of our approach (described in the following section). The *F* score is going to be used in order to compare the results obtained by our algorithm with previous related results. In some of the previous studies, it has not been possible to compute the *F* score from the published information and the recall or accuracy numbers are then presented.

The achieved results for our generative model applied to the five-participant database are presented in four sub-sections. The first three sub-sections will study the number of layers of the auto-encoders stacked using the “cutting with a knife” movement. The last sub-section will use the selected parameters in the first sub-sections to validate the results when detecting the “eating with a spoon” movement. A comparison of the results achieved when training the stack of auto-encoders with and without our generative model is also added to the first sub-section. 

### 5.1. Parameter Selection for a Single Layer Using the “Cutting” Movement

**Figure 4:**
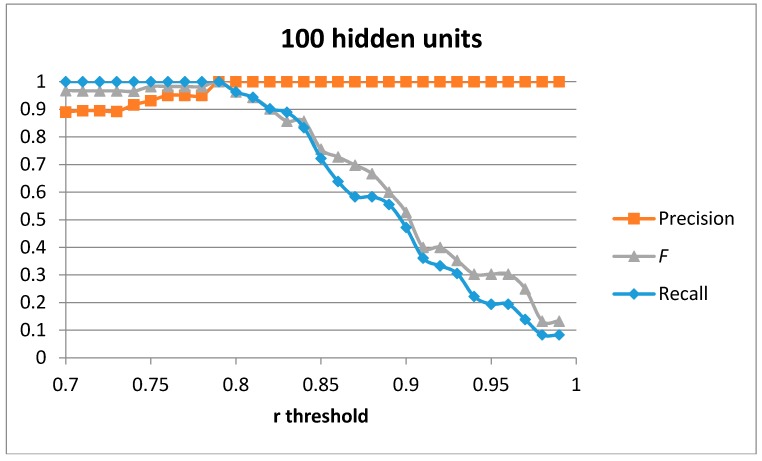
Precision, recall, and *F* score for 1 auto-encoder and 100 hidden units.

**Figure 5:**
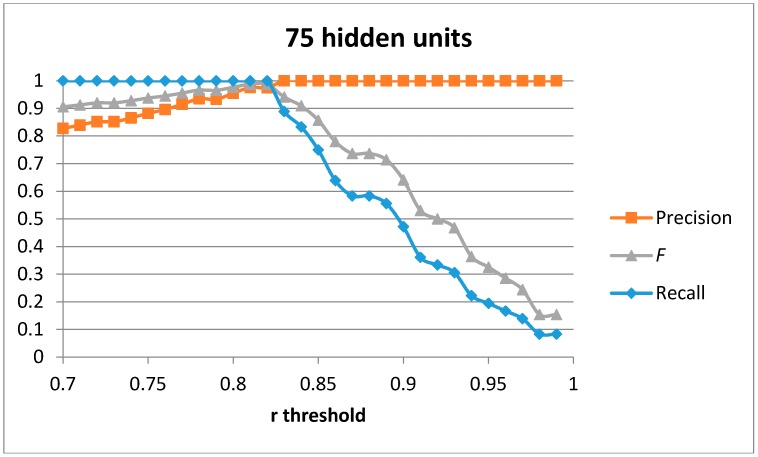
Precision, recall, and *F* score for 1 auto-encoder and 75 hidden units.

**Figure 6:**
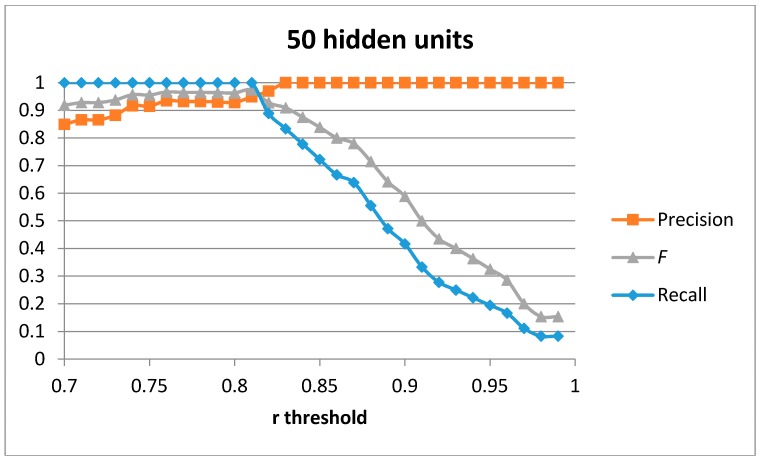
Precision, recall, and *F* score for 1 auto-encoder and 50 hidden units.

**Figure 7:**
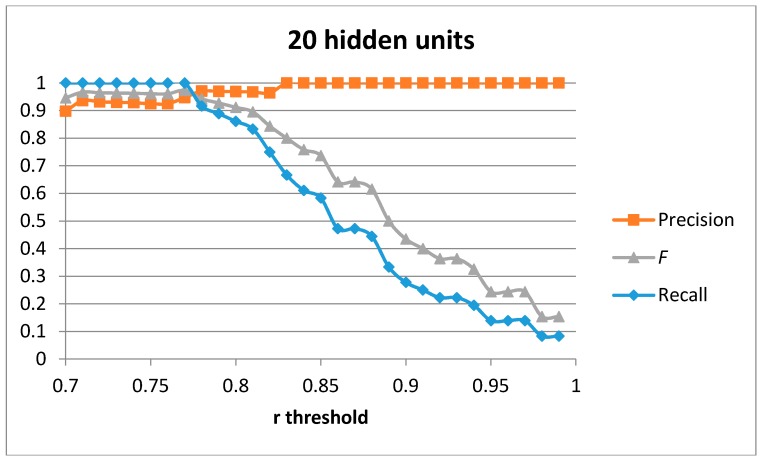
Precision, recall, and *F* score for 1 auto-encoder and 20 hidden units.

The first result set has been generated by training a single auto-encoder with the 51 generated samples for the “cutting” movement with different number of hidden units. A leave-one-out approach has been used to compute the results. Figure 4, Figure 5, Figure 6 and Figure 7 show the obtained results for 100, 75, 50 and 20 hidden units in the encode and decode functions of the auto-encoder (the input window of sensed data contains 200 samples, or 2 s of data). The threshold value for the Pearson correlation coefficient used to select a validation sample as a cutting or non-cutting movement is used as the independent parameter in each figure. For small values of the threshold, all cutting movements in the validation sequence are detected and the achieved recall value is optimal. However, some of the non-cutting movements are also classified as cutting movements and the precision of the algorithm worsens. The *F* score is computed for each value of the independent variable in order to compare each configuration. The best results are captured in Table 3. The results show that a single auto-encoder is able to achieve an *F* score of 1 when 100 hidden units are used. The performance slightly decreased if the number of hidden units is also decreased. However, even for the case of using 20 hidden units, the F score achieved is 0.947, which is similar to the best value in Table 2 for the research study in [18]. The threshold correlation coefficient has to be decreased when the number of hidden units is small (under 50) in order to achieve optimal results. An explanation for this is that the similarity of the reconstructed sample to the sensed data segment decreases as the degrees of freedom (the number of hidden units) in the algorithm decrease. 

**Figure 8:**
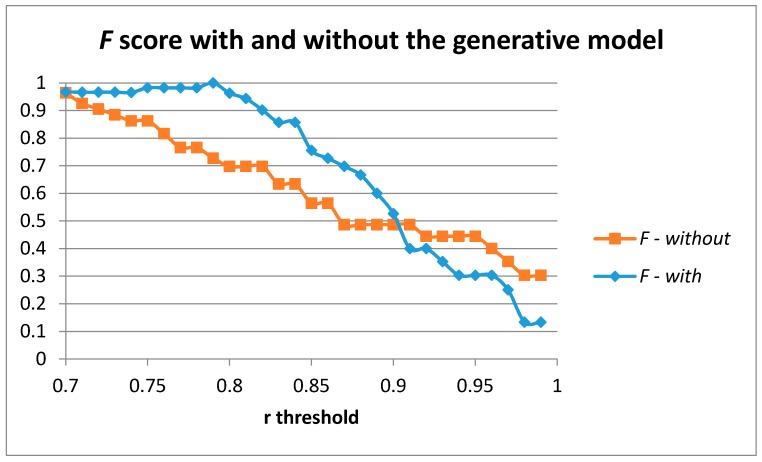
Results using the generative model compared to those achieved without the generative model for one layer of 100 hidden units in the auto-encoder.

Figure 8 captures the results achieved for the *F* score when moving the r threshold in the case of using and not using our generative model when training the auto-encoder with one hidden layer of 100 units. The use of the generative model provides a better description and characterization of the movement to be detected and provides better results compared to the use of the sensed samples without the generative model. 

### 5.2. Parameter Selection for Two Layers Using the “Cutting” Movement

Adding a second layer to the stack of auto-encoders increases the degrees of freedom and therefore the required size of the training data samples is also increased in order to avoid overfitting problems. Our generative model is able to generate as many different training samples at different speeds of execution as needed. However, in order to compare results with those presented in the previous sub-section, the same 51 training samples have been used. 

**Figure 9:**
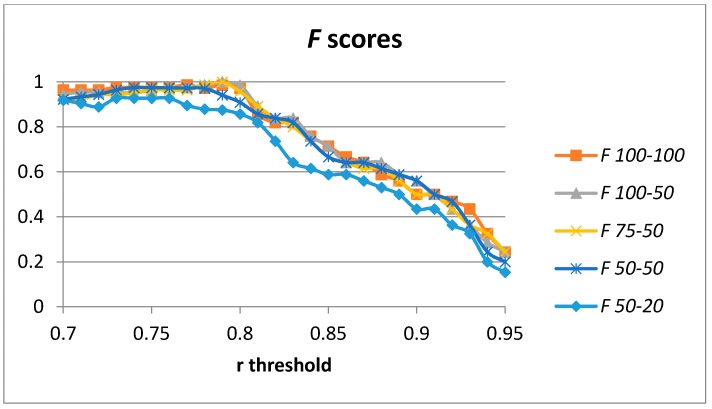
*F* scores for 2 stacked auto-encoders and 100-100, 100-50, 75-50, 50-50, and 50-20 hidden units.

Optimal results were achieved for 100 hidden units in the case of a single auto-encoder. We have computed the results for 100-100, 100-50, 75-50, 50-50, and 50-20 hidden units (in the first and second layers, respectively). Figure 9 captures the results for the *F* score considering the threshold for the Pearson correlation coefficient used in order to classify each validation sample into a cutting or non-cutting movement. The results for the optimal values for the *F* score for each configuration are captured in Table 4. In this case, optimal values are achieved by the 100-50 and the 75-50 configurations for the number of hidden units. The results for the 100-100 configuration are slightly worse (probably due to overfitting in the model). The 50-50 configuration is able to provide a value of *F* = 0.975, which is higher than the previous studies in Table 2. 

### 5.3. Parameter Selection for Three Layers Using the “Cutting” Movement

**Figure 10:**
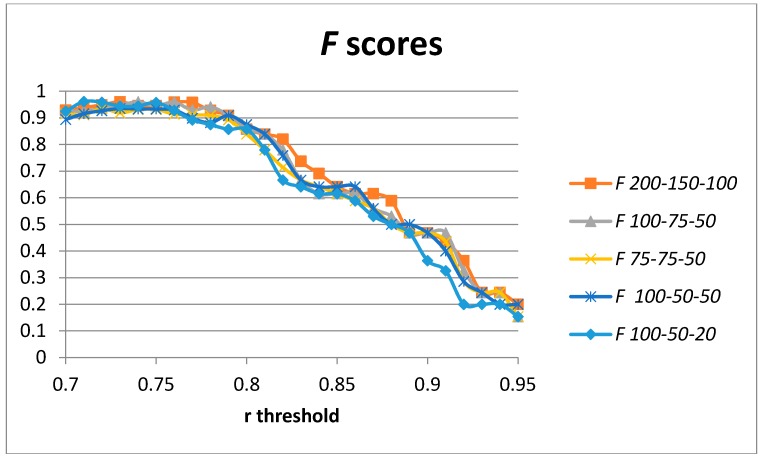
*F* scores for three stacked auto-encoders and 200-150-100, 100-75-50, 75-75-50, 100-50-50, and 100-50-20 hidden units.

We have added a third layer of auto-encoders to our model in order to assess the changes in the obtained precision and recall. A third layer of auto-encoders has not always provided better results [19]. The increase in the number of degrees of freedom tends to require a bigger number of training samples to avoid overfitting. We have used the same generated data sample in order to compare results with those presented in previous sections. The achieved results for the F score for different configurations in terms of the number of hidden units used in the stack of auto-encoders are captured in Figure 10. The optimal values for the F score for each configuration are captured in Table 5. Optimal values are achieved for the 200-150-100 and 100-75-50 hidden units. However, the results are worse than in the two-layer architecture. 

### 5.4. Detecting the “Eating with a Spoon” Movement

In this section, we are going to train a second stack of auto-encoders with the best parameters obtained in the previous sections in order to detect the “eating with a spoon” movement. Using a leave-one-out validation approach (using four users in order to train the stack of auto-encoders and one for validation), we first generate 142 equally spaced samples (with a constant increment in the speed of execution) using the generative model proposed in this paper. In order to pre-select and align candidate segments of acceleration data containing the “eating with a spoon” movement, the standard deviation on a 100-ms window centered at each point is first calculated. Candidate segments should have an “up” followed by a “down” gesture within a certain time span (taking into account the maximum and minimum speeds for the execution of the movement). “Up” and “down” movements are detected by analyzing the maximum values of the standard deviation data. The maximum value for the speed of execution of the movement (in our recorded data) corresponds to a time span of 480 ms. The minimum speed value for the speed of execution of the movement corresponds to a time span of 1900 ms. Pre-selected segments are aligned to the mean value between the “up” and “down” movements. Table 6 captures the recall, precision and *F* score for the best configurations for one and two layers of auto-encoders as analyzed in the previous sub-sections. The confusion matrix when using the best configuration is presented in Table 7. There are four gestures of eating out of 88 that are classified as “other”. There are three segments of other movements (performed while “walking” (2) and “free arm movements” (1)), which are classified as “eating with spoon”. The results outperform those in previous studies, as captured in Table 2, by at least 1.8 percentage points. 

## 6. Generalization of Results

In order to assess the generalization of results to different people using different hardware when performing a different experiment in which the “cutting with a knife” movement is also included, we have applied the auto-encoders trained in the previous section using the data generated in the previous section to the database in [20]. This human movement database recorded arm movements of two people performing a continuous sequence of eight gestures of daily living (including the “cutting with a knife” movement). The authors also recorded typical arm movements performed while playing tennis. In addition, they also included periods with no specific activity as the NULL class. The activities were performed in succession with a brief break between each activity. Each activity lasted between two and eight seconds and was repeated 26 times by each participant, resulting in a total dataset of about 70 min. Arm movements were tracked using three custom Inertial Measurement Units placed on top of each participant’s right hand, as well as on the outer side of the right lower and upper arm. Each Inertial Measurement Unit comprised a three-axial accelerometer and a two-axis gyroscope recording timestamped motion data at a joint sampling rate of 32 Hz [20]. We have only used the data from the accelerometer placed on top of the dominant hand since it is located in a similar position as the one we used to train the stack of auto-encoders in the previous section. The data was resampled at 100 Hz and windowed using 2-s segments of sensor data aligned to pre-selected points (local maximum values with an estimated execution speed of the captured movement in the expected range for the “cutting with a knife” movement). 

**Figure 11:**
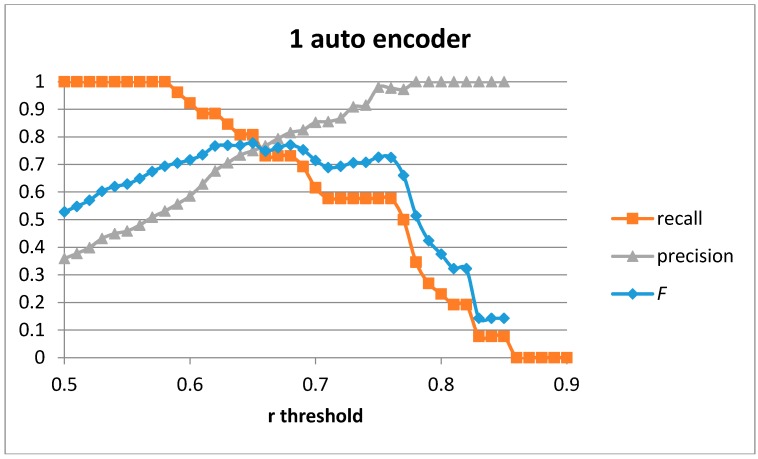
Precision, recall, and *F* score for 1 auto-encoder and 100 hidden units.

The best performing configuration for the number of hidden units for 1, 2, and 3 stacked auto-encoders have been used. Figure 11 shows the recall, precision, and *F* score for the case of a single auto-encoder with 100 hidden units. The best values obtained are for *F* = 0.77 (recall = 0.731 and precision = 0.816). The best values in [20] are *F* = 0.75 and only when using the three sensor devices and for the person-dependent case (training and validating using the data for the same person). Using only the hand sensor in a person-dependent validation the authors were only able to achieve *F* = 0.559. Validating the detection algorithm in a person-independent way (training with the data of one user and validating with the sensed data from the second user) provided *F* = 0.486 for the three sensors and *F* = 0.289 for the single hand sensor. 

In order to compare the results for the three configurations using one, two, and three layers of auto-encoders, the Area under the Curve (AuC) for the *F* score has been computed. This value will provide an estimation about how sensitive results are when non-selecting the optimal threshold value for the Pearson correlation coefficient. In the case of a single auto-encoder, the AuC = 0.217.

The authors in [20] present the confusion matrix for person-independent evaluation and all data for one of the participants. For the case of the “cutting with a knife” gesture, the achieved recall was 0.615 and the precision 0.781. In order to compare results, the classification results for a similar recall of 0.615 when using a single auto-encoder trained with our generative model are shown in Table 8. The precision achieved using our approach is 0.865. The number of correctly classified cutting gestures is also captured. 

Table 9 shows the classification results for the optimal *F* value. In this case, the optimal *F* score is 0.77 which is able to detect 73.1% of all the cutting gestures and provides a precision of 0.816. The majority of false positives are samples containing the stirring gesture, which is similar to the cutting gestures when the execution speeds are similar. Only 4.4% of false positives come from other gestures in the database.

**Figure 12:**
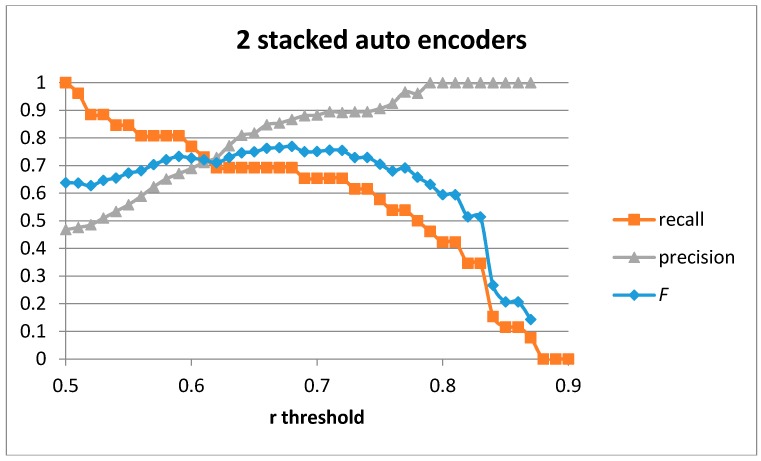
Precision, recall, and *F* score for two stacked auto-encoders and 100-50 hidden units.

Figure 12 shows the recall, precision, and *F* score for the case of two stacked auto-encoders with 100-50 hidden units. The best values obtained are for *F* = 0.77 (recall = 0.692 and precision = 0.867). The values are similar to those obtained for the case of a single auto-encoder. However, in the case of two stacked auto-encoders the AuC improves to 0.237. By using two auto-encoders, the results get less conditioned to the optimality in the threshold selection improving the robustness of the detection system and increasing the optimal parameter selection window.

The classification results for a recall of 0.615 when using two stacked auto-encoders trained with our generative model are shown in Table 10. The precision achieved using our approach improves to 0.889 compared to 0.865 for the case of a single auto-encoder (which is congruent with the increase in the AuC value). The number of false positives decreases. The number of correctly classified cutting gestures is also captured. 

Table 11 shows the classification results for the optimal *F* value. In this case, the optimal *F* score is 0.77, which is able to detect 69.2% of all the cutting gestures and provides a precision of 0.867. The majority of false positives are samples containing the stirring gesture, which is similar to the cutting gestures when the execution speeds are similar. Only 3.1% of false positives come from other gestures in the database.

**Figure 13:**
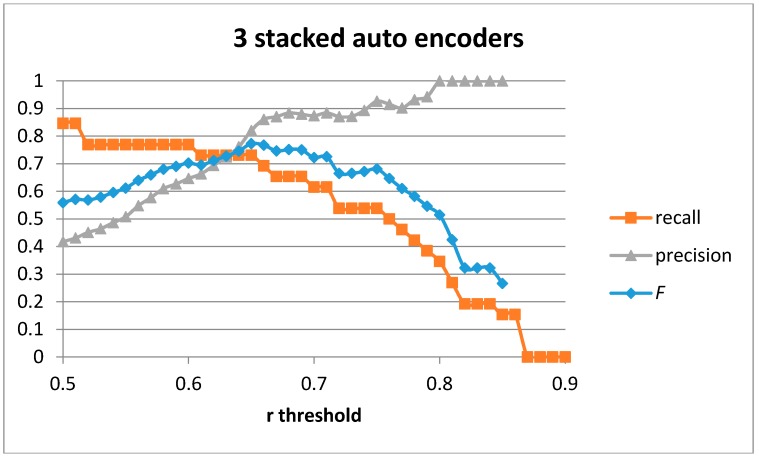
Precision, recall, and *F* score for three stacked auto-encoders and 100-75-50 hidden units.

Figure 13 shows the recall, precision, and *F* score for the case of three stacked auto-encoders with 100-75-50 hidden units. The best values obtained are for *F* = 0.77 (recall = 0.731 and precision = 0.82). The values are similar to those obtained for the case of a single or 2 stacked auto-encoders. However, in the case of three stacked auto-encoders, the AuC worsens to 0.22 compared to 0.237 when using two stacked auto-encoders. 

The classification results for a recall of 0.615 when using three stacked auto-encoders trained with our generative model are shown in Table 12. The precision achieved using our approach is 0.889 (the same as when using two auto-encoders). The number of correctly classified cutting gestures is also captured. 

Table 13 shows the classification results for the optimal *F* value. In this case, the optimal *F* score is 0.77, which is able to detect 73.1% of all cutting gestures and provides a precision of 0.82. The majority of false positives are samples containing the stirring gesture, which is similar to the cutting gestures when the execution speeds are similar. Only 2.2% of false positives come from other gestures in the database.

## 7. Conclusions

Deep learning techniques applied to human sensed data from wearable sensors have shown better results in previous research studies as compared with previous machine learning techniques in order to detect specific human movements and activities. However, using deep learning techniques, the degrees of freedom to be trained in the system tend to increase and so does the number of training samples required to avoid overfitting problems.

This paper proposes a movement detection approach using a deep learning method based on stacked auto-encoders applied to one of the axes of a single accelerometer placed on the wrist of the dominant hand. In order to simplify the data gathering for the training phase, a novel generative model based on the time elasticity of the human movements when performed at different speeds has been defined and validated. A simple pre-detection algorithm based on the statistical properties of some points of interest for each activity is added in order to pre-select candidate time windows and a time-offset cancellation mechanism (sequence alignment) is used by centering the selected data window based on the detected candidate points of interest. 

The results show that it is possible to achieve optimal results for detecting particular movements when using a database of different users following the same instructions and using the same sensors and procedures. Using the leave-one-out validation method for a database of five users performing six different movements, we achieve *F* = 1 when detecting the “cutting with a knife” movement. The results are able to generalize to a second database obtained from two different people using different hardware and a different recording procedure, executing other movements (cutting being one of them). The optimal results are obtained when using a two-layer architecture and a value of *F* = 0.77, which improves the best case in the original study using the same database (*F* = 0.75).

In the future, we plan to apply the proposed generative model to other human movements to confirm that results generalize to different human movements that are executed at different speeds.

## Figures and Tables

**Table 1:** Participant demographics.

Gender	Age	Height (m)	Weight (kg)
F	32	1.73	62
M	38	1.75	77
F	39	1.68	71
F	41	1.65	64
M	43	1.83	89

**Table 2:** Best results in similar previous studies.

Previous Related Studies	*F*/Recall/Acc.	Method	Database	Data Dimensionality
[21]	*F* = 0.927	b-LSTM-S ^1^	[22]	79
[21]	*F* = 0.937	CNN ^2^	[23]	52
[21]	*F* = 0.760	LSTM-S ^1^	[24]	9
[6]	*F* = 0.851	CNN ^2^	[22]	79
[25]	*F* = 0.917	DeepConvLSTM ^1^	[22]	79
[7]	Recall = 0.950	DRNN ^3^	[26]	3
[18]	*F* = 0.948	CNN ^2^	[18]	6
[19]	Accuracy = 0.856	SDAE ^5^	[19]	23
[20]	*F* = 0.750	NCC ^4^	[20]	9

^1^ LSTM = Long Short-Term Memory. ^2^ CNN = Convolutional Neural Network. ^3^ DRNN = Deep Recurrent Neural Network. ^4^ NCC = Nearest Class Centroid, ^5^ SDAE = Stack of two De-noising Auto-encoders.

**Table 3:** Optimal values for the *F* score for each configuration.

Values for the Optimal *F*	100	75	50	20
r threshold	0.8	0.8	0.81	0.77
*F*	1	0.988	0.974	0.973
Recall	1	1	1	1
Precision	1	0.976	0.949	0.947

**Table 4:** Optimal values for the *F* score for each configuration.

Values for the Optimal *F*	100-100	100-50	75-50	50-50	50-20
r threshold	0.77	0.79	0.79	0.74	0.76
*F*	0.987	1	1	0.975	0.928
Recall	1	1	1	1	0.889
Precision	0.974	1	1	0.951	0.97

**Table 5:** Optimal values for the *F* score for each configuration.

Values for the Optimal *F*	200-150-100	100-75-50	75-75-50	100-50-50
r threshold	0.76	0.76	0.75	0.75
*F*	0.959	0.959	0.932	0.933
Recall	0.972	0.972	0.944	0.972
Precision	0.946	0.946	0.919	0.897

**Table 6:** Values for the optimal parameters selected from previous sections.

Values for the Optimal *F*	100	100-50
r threshold	0.82	0.79
*F*	0.960	0.966
Recall	0.954	0.966
Precision	0.966	0.966

**Table 7:** Classification results for two layers of auto-encoders.

Classified as Ground Truth	Cutting with A Knife	Eating with Spoon	Other
Cutting with a knife	112	0	0
Eating with spoon	0	84	3
Other	0	4	208

**Table 8:** Classification results for one layer of auto-encoders and recall 0.615.

Classified as Ground Truth	Cutting	Other	Precision
Cutting	32	5	0.865
Other	20	572	
Recall	0.615		

**Table 9:** Values for the optimal *F* (*F* = 0.77, recall = 0.731, precision = 0.816).

All Positives	Cutting	Stirring	Other
Detected as cutting	0.816	0.14	0.044

**Table 10:** Classification results for two layers of auto-encoders and recall 0.615.

Classified as Ground Truth	Cutting	Other	Precision
Cutting	32	4	0.889
Other	20	572	
Recall	0.615		

**Table 11:** Values for the optimal *F* (*F* = 0.77, recall = 0.692, precision = 0.867).

All Positives	Cutting	Stirring	Other
Detected as cutting	0.867	0.102	0.031

**Table 12:** Classification results for three layers of auto-encoders and recall 0.615.

Classified as Ground Truth	Cutting	Other	Precision
Cutting	32	4	0.889
Other	20	572	
Recall	0.615		

**Table 13:** Values for the optimal *F* (*F* = 0.77, recall = 0.731, precision = 0.82).

All Positives	Cutting	Stirring	Other
Detected as cutting	0.82	0.158	0.022
